# Diosmetin Protects against Cardiac Hypertrophy via p62/Keap1/Nrf2 Signaling Pathway

**DOI:** 10.1155/2022/8367997

**Published:** 2022-02-22

**Authors:** Yingying Guo, Dan Li, Xian-feng Cen, Hong-liang Qiu, Yu-lan Ma, Yi Liu, Si-hui Huang, Li-bo Liu, Man Xu, Qi-Zhu Tang

**Affiliations:** ^1^Department of Cardiology, Renmin Hospital of Wuhan University, Wuhan 430060, China; ^2^Cardiovascular Research Institute of Wuhan University, Wuhan 430060, China

## Abstract

An important pathophysiological consequence of pressure overload-induced cardiac hypertrophy is adverse cardiac remodeling, including structural changes in cardiomyocytes and extracellular matrix. Diosmetin (DIO), a monomethoxyflavone isolated from citrus fruits, had antioxidative stress effects in multiple organs. The purpose of this study was to examine the biological effect of diosmetin on pathological cardiac hypertrophy. In mice, diosmetin treatment reduced cardiac hypertrophy and dysfunction in an aortic banding- (AB-) induced pressure overload model and reducing myocardial oxidative stress by increasing antioxidant gene expression. In vitro, diosmetin (10 or 50 *μ*m, 12 h or 24 h) protected PE-induced cardiomyocyte hypertrophy in neonatal rat cardiomyocytes. Mechanistically, diosmetin inhibited autophagy by activating the PI3K/Akt pathway. In particular, diosmetin induced the accumulation of p62 and its interaction with Keap1, promoted the nuclear translocation of Nrf2, and increased the expression of antioxidant stress genes in the process of cardiac hypertrophy. Furthermore, knockdown of p62 in rat primary cardiomyocytes abrogate the protective effect of diosmetin on cardiomyocyte hypertrophy. Similarly, the Nrf2 inhibitor ML385 obviously abolished the above effects by diosmetin treatment. In conclusion, our results suggest that diosmetin protects cardiac hypertrophy under pressure overload through the p62/Keap1/Nrf2 signaling pathway, suggesting the potential of diosmetin as a novel therapy for pathological cardiac hypertrophy.

## 1. Introduction

Cardiac hypertrophy is offset by temporary preservation of cardiac output, while persistent pathological cardiac hypertrophy is related to an increased risk of heart failure, arrhythmias, and sudden death [1, 2]. Although treatment choices have considerably improved prognosis and quality of life of patients diagnosed with heart failure, current therapy has at best put off disease progression, rather than providing a curative effect. Experimental evidence supports oxidative stress as a key causal factor in cardiac hypertrophy [3], a finding reinforced by elevated levels of circulating markers of oxidative stress in patients with cardiac hypertrophy [4]. Antioxidants have been shown to exert a protective effect in several animal models of cardiac hypertrophy [5]. In addition to traditional mediators of oxidative stress, dysregulation of autophagy and protein homeostasis also contribute to pathological cardiac hypertrophy through mechanisms involving oxidative stress [6, 7].

It is widely accepted that autophagy is dysregulated under hemodynamic stress [8]. In this sense, autophagy seems to be an attractive therapeutic target for cardiac diseases. The PI3K/Akt pathway is involved in cardiac hypertrophy by two well-established downstream proteins, mTOR and glycogen synthase kinase-3 (GSK-3), both of which modulate cardiomyocyte autophagy [9–11]. p62 (sequestosome-1/SQSTM1) serves as a selective autophagy receptor as well as a signaling scaffold to participate in the regulation of multiple physiological processes including oxidative stress defense and cellular metabolism [12–14]. p62 bodies can sequester Keap1, an adaptor of the cullin-3 E3-ubiquitin ligase complex for Nrf2. This uncouples Nrf2 from regulation by the ubiquitin-proteasome system (UPS), leading to its stabilization, followed by translocation to nucleus where it activates antioxidant target genes such as superoxide dismutase1 (SOD1), glutathione transferase, glutamate cysteine ligase and heme oxygenase 1 (HO-1) [15]. The Keap1-Nrf2 antioxidant pathway was found to be closely associated with pathological remodeling [16, 17]. However, the precise role and effective intervention of the p62-Keap1-Nrf2 pathway in pathological cardiac hypertrophy especially in pressure-overload heart remains elusive.

Traditional Chinese medicines have been widely and effectively used in treating cardiovascular diseases, and compounds from various herbs play important roles in taming diverse pathophysiological processes such as peroxidation and metabolic abnormalities [18]. The pharmacological effects of diosmetin have been recognized to possess anticancer, antimicrobial, antioxidant, and anti-inflammatory activities. Recently, it has been reported that diosmetin exerts an antitumor activity by upregulating ROS levels and inhibiting Nrf2 [19, 20]. In a model of hypoxia-injured myocardial cells established by using H9C2 cell line, diosmetin regulates autophagy through activation of AMPK signaling pathway, thereby inhibiting myocardial cell apoptosis [21]. Another study also points out diosmetin triggered autophagy by regulating the mTOR pathway in HepG2 cells [22]. Diosmetin modulates glucose metabolism by upregulating the IRS/PI3K/AKT signaling pathway to promote glycogen synthesis and GLUT4 translocation on T2DM [23].

Previous study has shown that diosmetin could ameliorate LV (left ventricular) dysfunction and remodeling and decrease oxidative stress in rats fed with a high-fat (HF) diet [24]. However, thus far, it is unclear whether diosmetin can improve the cardiac function of pressure overload-induced cardiac hypertrophy. Along this line, we have reasons to speculate that diosmetin can be used as a new drug to alleviate LV dysfunction induced by aortic banding. In this study, we confirmed that diosmetin protect against cardiac hypertrophy by regulating autophagy, leading to abnormal accumulation of p62 protein, activating p62-Keap1, and promoting Nrf2 translocation to nucleus to exert antioxidant effects. Localization of Nrf2 to the nucleus also allows an upregulation of the p62 expression. Thus, a positive feedback loop exists between Nrf2 and p62, which may explain the cardiac protective effect of diosmetin under pressure overload. We first determined the role of diosmetin in cardiac hypertrophy, explored its mechanism of action from the perspective of autophagy and antioxidation, and indicated diosmetin modulating the cellular localization of Nrf2 without altering the expression level of Nrf2.

## 2. Materials and Methods

### 2.1. Animals

All male C57BL/6 mice, 8-10 weeks old, weighing 25 ± 1 g, were purchased from the Institute of Laboratory Animal Science, Chinese Academy of Medical Sciences. Based on the National Institutes of Health's Guide for the Care and Use of Laboratory Animals, all animal experiments were approved by the Animal Care and Use Committee of the Wuhan University People's Hospital (the ethics approval number: SYXK 2020-0053).

Aortic banding (AB) surgery was performed as previously described [6]. Briefly, after anaesthetizing the mice with 3% pentobarbital sodium at 40 mg/kg, a 27G needle was used to ligate the thoracic aorta. The sham surgery was treated in the same way without ligation. The following day, diosmetin (Dio, 98% purity), purchased from Shang hai Winherb Medical Science Co., Ltd. (Shanghai, China), was given with 40 mg/kg or an equivalent volume of vehicle (0.1% DMSO PBS) by gavage [24]. At 4 weeks after sham or AB surgery, mice were cervically dislocated and executed, and the heart tissue was taken for further experiments.

### 2.2. Echocardiography

Before being sacrificed, mice were anaesthetized with 1.5% isoflurane and placed in the prone position, their chest hair was removed with hair removal cream, and transthoracic echocardiography was performed. Cardiac function was recorded using a MyLab 30CV ultrasound system (bisound Esaote, Florence, Italy) [25]. Left ventricular posterior wall (LVPW) and interventricular septum (IVS) thicknesses were measured during diastole. Short-axis M-mode images were captured at the papillary muscle level to record the internal dimensions of the left ventricle. Three consecutive cardiac cycle images were used to analysis shortening fraction and ejection fraction.

### 2.3. Isolation and Culture of Neonatal Rat Ventricular Myocytes

Briefly, hearts were extracted from newborn Sprague-Dawley rats within 3 days of birth and digested in collagenase-II (Worthington Biochemical, Lakewood, NJ, USA) and trypsin (Sigma-Aldrich, Taufkirchen, Germany) solutions. After about 12 times of digestion, cells were then separated by centrifugation in a Percoll gradient. NRCMs were obtained from the lower phase and seeded with medium consisting of DMEM (Biochrom, Berlin, Germany) supplemented with 10% fetal bovine serum (FBS) (Serana Europe, Pessin, Germany). Cardiomyocytes were cultured in 6-well plates for 24 h and in fresh DMEM supplemented with 10% FBS for 2-3 days before in vitro assays. Hypertrophy was induced by incubating with 25 *μ*mol/L phenylephrine (PE), with or without different concentrations of diosmetin (dissolved in 0.1% DMSO PBS at a concentration of 10 and 50 *μ*M) and incubated for further 12 or 24 h.

### 2.4. Cell Transfection

Lipofectamine 6000 reagent (Beyotime Company, C0526), p62 targeting siRNA and DMEM were made into a suspension and then transfected with NRVMs for 48 hours. Nontargeting control siRNA was used as a control. For mCherry-EGFP-LC3B adenoviral particles transfection, 24 hours posttransfection, the adenovirus was removed and cells were used for processing and confocal imaging 2 days posttransfection.

### 2.5. Immunocytochemistry

The NRCMs were fixed in 4% paraformaldehyde for 15 min at room temperature and then permeabilized with PBS containing 0.3% Triton X-100 for 20 min. Next, samples were blocked with 10% goat serum (GTX27481; GeneTex, Sanantonio, TX, USA) for 30 min and then incubated overnight at 4°C with the indicated primary antibodies. The next day, NRCMs were incubated with Alexa-Fluor-conjugated secondary antibodies (ZSGB-BIO,1 : 200) for 2 hours at room temperature. Anti-*α*-actinin (Cell Signaling, 69758S, 1 : 200) was used to measure the size of NRCMs. Cell colocalization analysis was performed using anti-Keap1 and anti-p62 coincubation and observed under the confocal microscope (TCS-SP8, Leica). DNA was stained with DAPI (1 *μ*M in PBS).

### 2.6. Measurement of Intracellular Reactive Oxygen Species (ROS)

NRCMs were incubated with 10 *μ*m/L DCFH-DA (Beyotime Company, S0033S) in PBS for 30 minutes and washed three times with PBS. Then, the NRCMs were imaged on a fluorescence microscope (Olympus IX51) and average DCF fluorescence per cell was determined with ImageJ. Dihydroethidium (DHE, Sigma-Aldrich, USA) was used to detect the superoxide level in vivo. According to the manufacturer's protocol, frozen sections were incubated with 10 *μ*M DHE for 20 min at room temperature. Then, the images were captured using a fluorescence microscope (OLYMPUS DX51, Tokyo, Japan) and quantified by analyzing fluorescence intensity using ImageJ.

### 2.7. Primers Used in Quantitative Real-Time PCR (qPCR)

Total RNA was extracted from NRCMs and heart tissue using TRIzol reagent (Invitrogen, Carlsbad, USA) and reversed transcribed into complementary DNA for qPCR. The LightCycler 480 SYBR Green 1 Master Mix (04887352001, Roche) was used for qPCR with the following thermal cycling parameters: 95°C for 15 s, 55°C for 15 s, and 72°C for 15 s for 40 cycles. Relative gene expression levels were normalized to the reference gene GAPDH using the *ΔΔ*CT method. Primer sequences for real-time PCR are shown in the supplementary data table 1.

### 2.8. Western Blot

Heart tissue was lysed and subjected to 10%-12%SDS-PAGE gels and transferred to PVDF membranes. Membranes were blocked with 5% skim milk at room temperature for 60 minutes and incubated overnight at 4°C with one of the following antibodies: GAPDH, LC3I/II, p62, Beclin-1, p-AKT, AKT, Nrf2, Keap1, and ATG7 (Cell Signaling, catalog numbers 2118, 12741, 13121s, 3495s, 4060, 4691, 12721, 4678, and 2631s), HO-1, SOD2, and Lamin B1 (Abcam, catalog number ab-13243, ab38155, and ab16048). After rinsing, membranes were then incubated with HRP-conjugated secondary antibody and visualized using a chemiluminescence system (Bio-Rad Laboratories, Inc.). Nuclear extracts were prepared using the Nuclear Extraction Kit (Beyotime Company, P0027), according to the manufacturer's instructions.

### 2.9. Statistical Analysis

All data are presented as the mean ± SEM. Differences between groups was assessed by *t*-test or one-way ANOVA using GraphPad Prism version 8.0. A value of *p* < 0.05 was considered to be statistically significant.

## 3. Results

### 3.1. Diosmetin Attenuates Cardiac Hypertrophy in Pressure Overload Hearts

**Figure 1:**
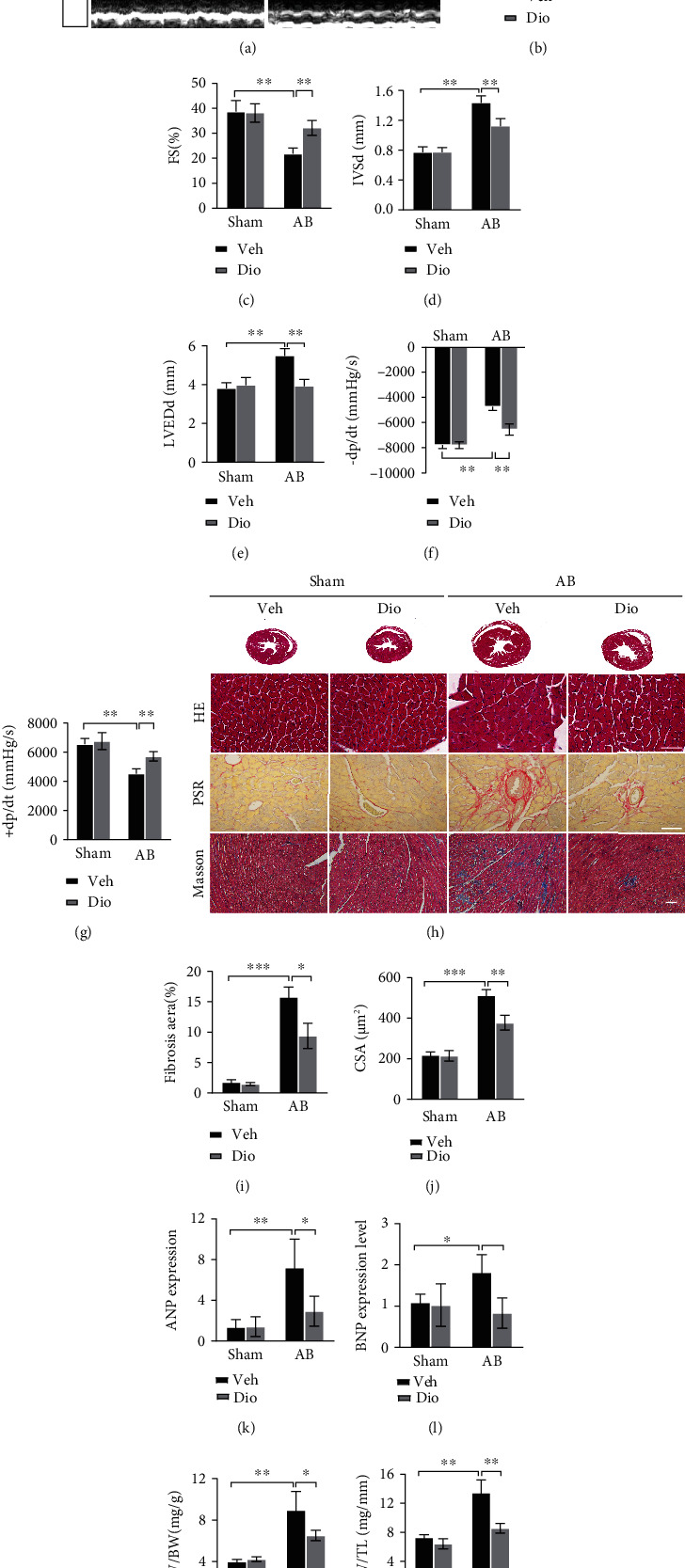
Improvement of cardiac function at 4 weeks after AB by diosmetin treatment. (a) Representative M-mode images. Echocardiography was performed to measure LVEF (b), LVFS (c), IVSd (d), and LVEDd (e), n =6. (f, g) Hemodynamic parameters in suggested groups (n = 6). (h) Histopathological images of heart tissue representing Cross-sectional views of mouse hearts and the cross-sectional area of cardiomyocytes stained by HE (scale bar: 50 *μ*m). Perivascular collagen synthesis stained by picrosirius red (PSR) (scale bar: 50 *μ*m), and myocardial fibrosis stained by Masson trichrome staining(blue areas indicate fibrosis, scale bar: 100 *μ*m), respectively. Quantitative of HE staining (n = 80–100) (j) and PSR staining (n = 3) (i). mRNAs for cardiac hypertrophy-associated genes ANP (k) and BNP (l) were measured by qPCR (n = 6). Gravimetric analysis of heart weight/body weight ratio (HW/BW) (m) and heart weight/tibia length (HW/TL) (n), n =6. Data are presented as mean ± SEM. ∗p <0.05, ∗∗p < 0.01, and ∗∗∗p < 0.001.

In order to analyze the effect of diosmetin on cardiac function, WT mice were subjected to aortic banding (AB) surgery with or without diosmetin treatment. We show that diosmetin treatment have improved cardiac function, as indicated by upregulated contractility (Figure 1(a)), ejection fraction (EF, Figure 1(b)), fractional shortening (FS, Figure 1(c)), interventricular septal thickness at the end-diastole (IVSd, Figure 1(d)), and LV internal diastolic diameter (LVIDd, Figure 1(e)) compared to AB controls. Consistently, compared to the AB group, the diosmetin-treated mice showed an increased rate of pressure development or decay (±dp/dt) (Figures 1(f) and 1(g)). Other ultrasound parameters that represent cardiac function in mice have been listed in the supplementary data table 2. There was no apparent difference among sham groups with or without diosmetin treatment. Moreover, the hearts from diosmetin treatment mouse that underwent AB were significantly smaller than those from AB controls (Figure 1(h)); these differences can also be seen in the heart weight to body weight (HW/BW) ratios (Figure 1(m)) and heart weight to tibia length (HW/TL) ratios (Figure 1(n)). Cross-sectional views of mouse hearts stained by HE staining(Figure 1(h)) show increased cross-sectional area of cardiomyocytes in response to diosmetin after AB surgery (Figure 1(j)). Picrosirius red (PSR) and Masson's trichrome staining (Figure 1(h)) suggested diosmetin prevented increased fibrogenesis both in the myocardium and perivascular area (Figure 1(i)). Consistently, classic markers of cardiac hypertrophy, such as atrial natriuretic peptide (ANP) and brain natriuretic peptide (BNP) (Figures 1(k) and 1(l)) downregulated in the hearts of diosmetin treatment mice after AB in comparison to AB controls.

## 4. Diosmetin Dose Dependently Ameliorated Cardiomyocyte Hypertrophy in Response to Phenylephrine

**Figure 2:**
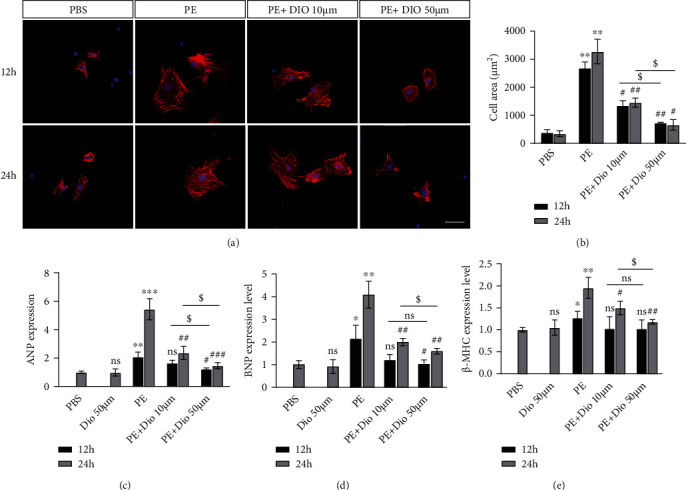
The impact of diosmetin on cardiomyocyte hypertrophy induced by PE. (a) Representative immunofluorescent images of NRVM stained with sarcomeric *α*-actinin (green) and DAPI (blue) and quantification of NRVM size (b), scale bar: 50 *μ*m, n = 3. (c–e) Quantitative real-time PCR analysis mRNA expression of hypertrophic markers ANP, BNP, and *β*-MHC. Data are presented as mean ± SEM. ∗p <0.05, ∗∗p < 0.01, and ∗∗∗p < 0.001 vs. the PBS group; #p <0.05, ##p < 0.01, and ###p < 0.001 vs. the PE (12 h) group; $p <0.05, $$p < 0.01, and $$$p < 0.001 vs. the PE (24 h) group; §p <0.05 vs. the PE+Dio group; ns: not significant.

Furthermore, neonatal rat ventricular cardiomyocytes (NRCMs) were isolated and treated with phenylephrine (PE) for 12 or 24 h to induce hypertrophy. In line with the above experimental studies, *α*-actinin immunostaining of NRCMs showed that diosmetin significantly decreased the cardiomyocyte size after 12 and 24 h cocultured with PE in a dose-dependent manner (Figures 2(a) and 2(b)). Compared to the only PE-treated group, the induction of ANP, BNP, and *β*-myosin heavy chain (*β*-MHC) transcription by PE for 24 h was clearly suppressed in the presence of diosmetin in a dose-dependent manner ranging between 10 and 50 *μ*M, whereas the treatment with PE for 12 h in the presence of diosmetin did not reach statistical significance (Figures 2(c) and 2(e)). The above results showed that pretreatment with 50 *μ*M diosmetin for 24 h provided maximal protection compared to lower concentrations. Therefore, 24 h pretreatment of diosmetin at 50 *μ*M was selected for subsequent experiments in the cardiomyocyte hypertrophy model. Taken together, our data suggest diosmetin attenuates cardiac hypertrophy both in vivo and in vitro.

## 5. Diosmetin Restrained Cardiac Oxidative Stress

**Figure 3:**
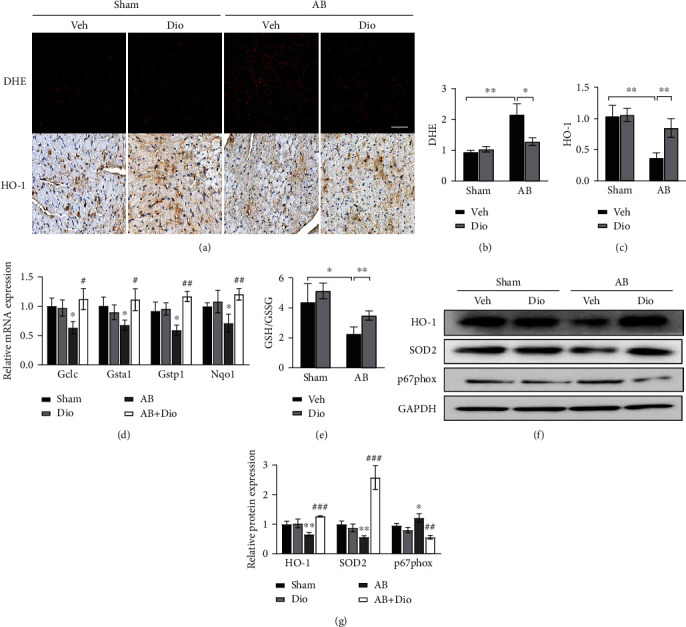
ROS reduction contributes to the protective effect of diosmetin in vivo. (a) Dihydroethidium (DHE) staining and cardiac expression of HO-1 four weeks after AB detected by immunohistochemistry, scale bar: 50 *μ*m. Quantitative analysis of myocardial ROS levels (b) and the expression of HO-1 (c), n = 3. (d) mRNA of antioxidant enzymes Gclc, Gsta1, Gstp1, and Nqo1 mRNA in whole ventricular lysates as measured by qPCR. (e) GSH/GSSG ratios in cardiac tissues, n = 4. (f, g) Levels of HO-1, SOD2, p67phox, and GAPDH were analyzed by Western blots using GAPDH as a loading control in heart tissue lysates. Data are presented as mean ± SEM. ∗p <0.05, ∗∗p < 0.01 vs. the sham group; #p <0.05, ##p < 0.01, and ###p < 0.001 vs. the AB group; ns: not significant.

Previous studies have shown that diosmetin has been identified possessing antioxidant activity in many disease models; hence, we evaluated the antioxidant effects of diosmetin in pressure overload hearts. DHE assay showed that the level of overall superoxide anion radicals was increased significantly in the AB group, which was reduced after diosmetin pretreatment (Figures 3(a) and 3(b)). Immunohistochemical staining of HO-1 also suggested an antioxidant activity of diosmetin in cardiac hypertrophy (Figures 3(a) and 3(c)). Moreover, real-time PCR indicated that diosmetin significantly increased the mRNA levels of Gstp1, Gclc, Gsta1, and Nqo1 (Figure 3(d)) in the AB group. Reducing glutathione (GSH) and oxidized glutathione disulfide (GSSG), the primary function of which is to scavenge metabolically derived reactive oxygen species (ROS), were also detected in mice by the GSH and GSSG Assay Kit (Beyotime Company, S0053). The result showed a decline in the GSH/GSSG ratio in diosmetin-treated mice (Figure 3(e)). Protein expressions of HO-1 and SOD2 induced by diosmetin treatment in AB were significantly increased, while the expressions of p67phox decreased (Figures 3(f) and 3(g)). These data indicated that diosmetin inhibits oxidative stress by promoting the expression of antioxidant gene.

## 6. Diosmetin Manipulating p62-Keap1-NRF2 Signaling Pathway

**Figure 4:**
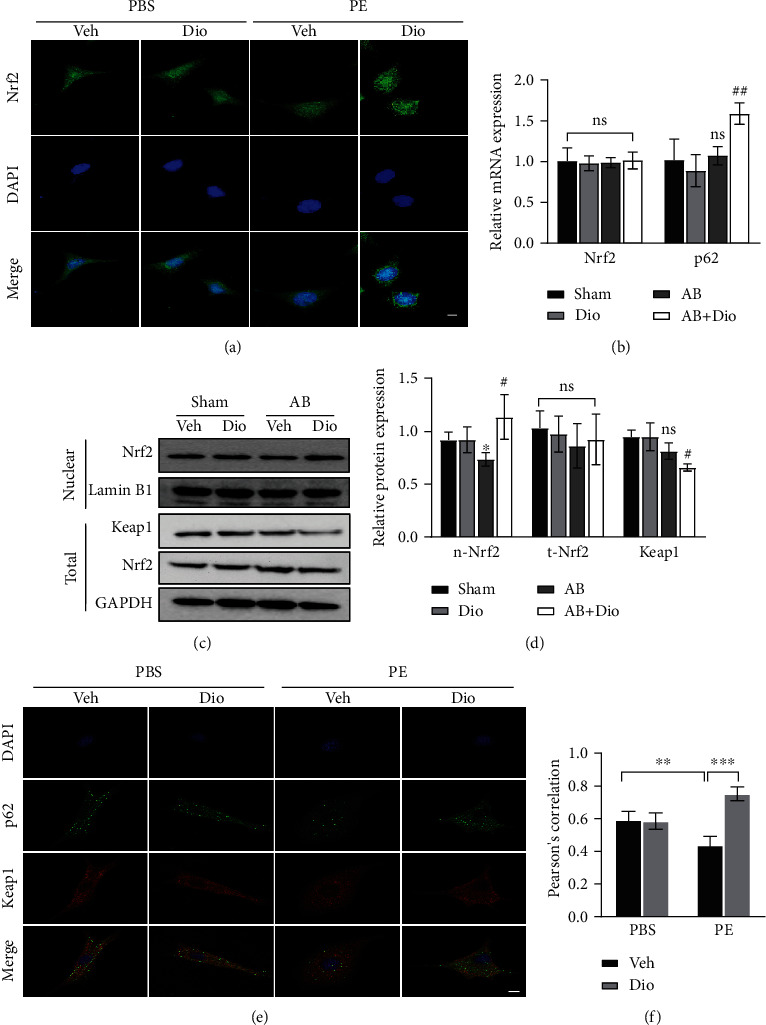
NRF2 signaling pathway is activated in response to diosmetin both in vivo and in vitro. (a) Representative immunofluorescence images of cells stained for Nrf2 (green) and nuclei with DAPI (blue), scale bar: 20 *μ*m, n = 3. (b) Level of mRNA analysed by real-time PCR in vivo, n = 3. (c, d) Representative western blots of Nrf2, Keap1, Lamin B1, and GAPDH protein level. (e) Representative confocal images of p62 (green) and Keap1 (red) signal colocalization and nuclei stained with DAPI (blue), scale bar: 10 *μ*m. (f) Quantitative analysis of confocal images, n =6. Data are presented as mean ± SEM. ∗p <0.05, ∗∗p < 0.01, and ∗∗∗p < 0.001 vs. the sham group, #p <0.05, ##p < 0.01 vs. the AB group; ns: not significant.

We observed that there was no difference of mRNA level of NRF2 in the AB group with or without diosmetin treatment (Figure 4(b)). However, the mRNA level of Nrf2 target genes, such as NQO1, HO-1, and SOD2 were upregulated. To further investigate the activation of oxidation resistance pathway in diosmetin heart during cardiac remodeling, we assuming that NRF2 cellular localization is altered. Western blot (Figures 4(c) and 4(d)) and immunofluorescence assay of Nrf2 in NRCMs (Figure 4(a)) revealed that diosmetin significantly increased Nrf2 nuclear translocation. And western blot also showed the total level of Nrf2 remained the same, whereas the protein level of Keap1 reduced in response to diosmetin in mice undergoing AB surgery. Notably, immunofluorescence staining assay showing that p62-Keap1 colocalization was significantly increased by diosmetin treatment in PE-induced cardiomyocyte hypertrophy (Figures 4(e) and 4(f)). In general, aggregation of p62 could recruit and sequester Keap1 to autophagosomes for degradation and consequently, Nrf2 is released freely and translocated to the nucleus where it activates transcription of antioxidant genes.

## 7. Diosmetin Inhibits PI3K/Akt Pathway-Dependent Autophagy

**Figure 5:**
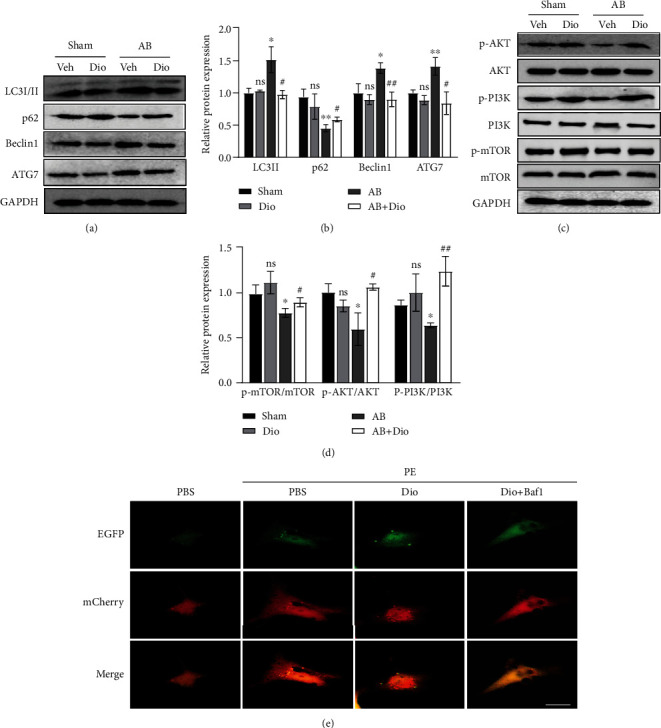
Diosmetin inhibited autophagy by enhancing the PI3K/Akt signaling pathway. (a) Representative western blot of autophagy-related protein LC3I/II p62/SQSTM1, Beclin1, and ATG7 in the heart. (c) Representative western blot of p-AKT, AKT, p-PI3K, PI3K, p-mTOR, mTOR, and GAPDH protein levels in the heart. (b, d) Quantitative of (a) and (c), respectively. (e) Representative confocal images showing colocalization of mCherry and EGFP signals in NRCMs transiently transfected with mCherry-GFP-LC3B adenovirus and treated as described above (one of 4 independent experiments), scale bar: 20 *μ*m. Baf1: Bafilomycin A1. Data are presented as mean ± SEM. ∗p <0.05, ∗∗p < 0.01 vs. the sham group; #p <0.05, ##p < 0.01 vs. the AB group; ns: not significant.

Considering that p62 aggregates could recruit Keap1 and activate autophagic degradation of Keap1, we then examined whether diosmetin regulates cardiac autophagy. Given that diosmetin regulates autophagy in hypoxia-injured H9C2 cell line, we questioned whether it also involved in the pressure-overloaded heart. Autophagy effectors, including LC3II, Beclin-1, ATG7, and p62/SQSTM1, were examined by Western blot (Figures 5(a) and 5(b)). The protein level of LC3II, Beclin-1, and ATG7 was higher in mouse subjected to aortic banding. However, the expression of LC3II, Beclin-1, and ATG7 was increased in response to diosmetin. Consistent with an increase in autophagy, p62/SQSTM1, a polyubiquitin-binding protein that is degraded during autophagy, showed the reverse trend. PI3K/Akt pathway, which modulates cardiomyocyte autophagy, is involved in cardiac hypertrophy. So, we next detected the PI3K/Akt signaling pathway. Compared with the AB group, the western blot results showed that diosmetin decreased the phosphorylation levels of Akt, PI3K, and mTOR in pressure-overloaded heart, indicating that diosmetin could activate the PI3K/Akt signaling pathway-mediated inhibition of autophagy (Figures 5(c) and 5(d)).

To confirm this assumption, we monitored the autophagic flux with a mCherry-EGFP-LC3B adenovirus. In control cells, we found PE-treated NRCMs showed an increase in the number of LC3B puncta with predominance of red ones. As expected, diosmetin led to both red and green LC3B puncta that exhibited yellow fluorescence in the cytoplasm, indicating that the autophagic flux is blocked. Bafilomycin A1 (BafA1) was used as a positive control due to its effectiveness in blocking the autolysosomal degradation pathway (Figure 5(e)).

## 8. Diosmetin Protect against Cardiomyocyte Hypertrophy in a p62-Dependent Manner

**Figure 6:**
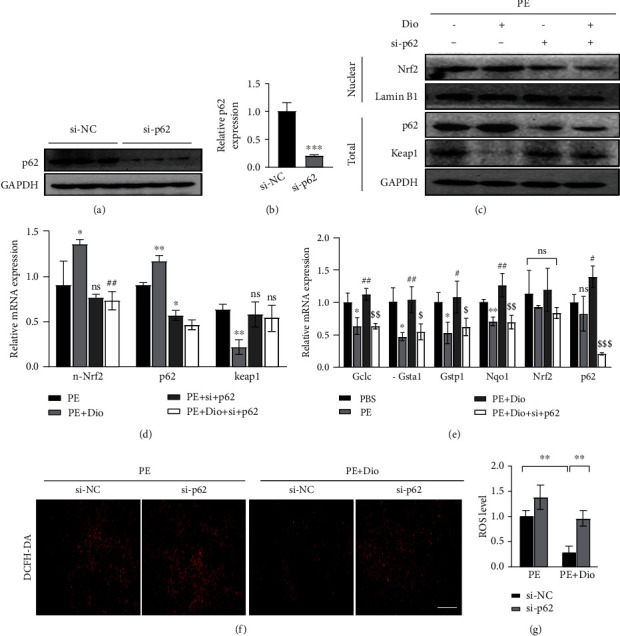
Knocking down of p62 in NRCMs suppresses Nrf2 signaling pathway in response to diosmetin. (a) Western blots of p62 in NRMCs transfected with control (si-NC) siRNA or p62 siRNA (si-p62) for 48 h. (b) Quantitative of the protein level of p62. The statistical significance was evaluated by Student's t-test; ∗∗∗p < 0.001. (c) Western blotting for Nrf2, Keap1, Lamin B1, p62, and GAPDH protein levels in isolated NRCMs infected with indicated siRNAs. (d) Quantification of (c); GAPDH was used as a loading control, ∗p <0.05, ∗p < 0.01 vs. the PE group; #p <0.05, ##p < 0.01 vs. the PE+Dio group. (e) Effect of diosmetin on the mRNA level of antioxidant enzymes Gclc, Gsta1, Gstp1, and Nqo1 in NRCMs and mRNA level of Nrf2 and p62 as measured by qPCR, n = 6, ∗p <0.05, ∗∗p < 0.01 vs. the PBS group; #p <0.05, ##p < 0.01 vs. the PE group; $p <0.05, $$p < 0.01, and $$$p < 0.001 vs. PE+Dio. Representative fluorescent images (f) and quantitative analysis of ROS by ImageJ (g), scale bar: 50 *μ*m. Data are presented as mean ± SEM, ∗∗p < 0.01. ns: not significant.

Since p62 has been implicated involving in autophagy as well as the Nrf2 signaling pathway, p62 was selected as a target to exploring further molecular mechanisms. We found that diosmetin treatment resulted in significant p62 accumulation. Therefore, we wondered whether knocking down p62 would reverse the effect of diosmetin. NRCMs were transfected with p62-siRNA or control-siRNA for 24 h, followed by diosmetin and PE treatment for 24 h. Western blotting analysis showed that protein levels of p62 were reduced by about 80% in siRNA-p62-transfected cells (Figures 6(a) and 6(b)). Consistent with the in vivo data presented in (Figures 4(c) and 4(d)), diosmetin treatment induced a significant reduction of Keap1 protein in PE-induced cardiomyocyte hypertrophy, but Keap1 protein degradation was blocked in siRNA-p62-transfected NRCMs (Figures 6(c) and 6(d)). Accordingly, diosmetin-triggered nuclear translocation of Nrf2 in cardiomyocytes was largely blocked by knockdown of p62 (Figures 6(c) and 6(d)) and the mRNA level of antioxidant gene such as Gstp1, Gclc, Gsta1, and Nqo1 also decreased after p62-siRNA transfection (Figure 6(e)). As a result, ROS was greatly reduced in NRCMs co-treated with diosmetin and PE compared to PE treatment alone (Figures 6(f) and 6(g)). However, when p62 expression was knocked down, such reduction was remarkably inhibited in NRCMs cotreated with diosmetin and PE (Figures 6(f) and 6(g)). These in vitro data further indicate that p62 is crucial for diosmetin-mediated protective effects against oxidative stress-induced cardiomyocyte damage.

## 9. Blockade of Nrf2 Activation by ML385 Suppresses Diosmetin-Mediated Protective Effects against PE In Vitro

**Figure 7:**
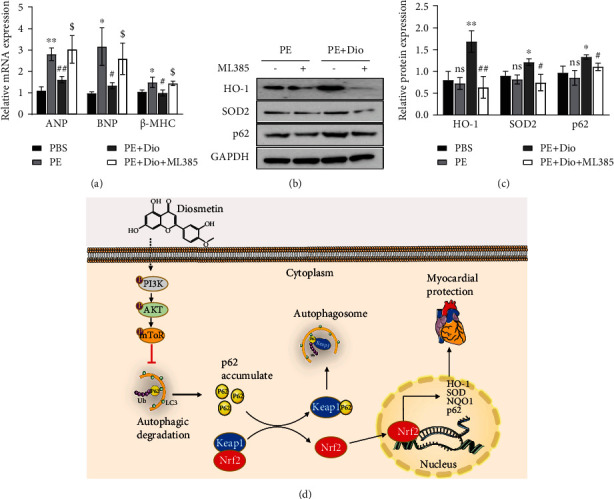
ML385 abolishes the cardioprotective effects of diosmetin in vitro. (a) qPCR analysis of mRNA of hypertrophy-related genes (ANP, BNP, and *β*-MHC) in NRCMs following stimulation with ML385, n = 3, ∗p <0.05, ∗∗p < 0.01 vs. the PBS group; #p <0.05, ##p < 0.01 vs. the PE group; $p <0.05 vs. PE+Dio. (b) Western blot of HO-1, SOD2, and p62 expressions in NRCMs with GAPDH as the control. (c) Quantitative analysis of (b), ∗p <0.05, ∗∗p < 0.01 vs. the PE group; #p <0.05, ##p < 0.01 vs. PE+Dio. Data are presented as mean ± SEM. ns: not significant. (d) A mechanism model diagram for the anticardiac hypertrophy effect of diosmetin.

ML385, a newly identified chemical compound that specifically binds to the Neh1 domain of Nrf2 and inhibits its downstream target gene expression, as well as reduces its own expression, was used to determine the significance of Nrf2 in diosmetin-mediated cardioprotection in vitro. The results showed that ML385 offset the antihypertrophic effects of diosmetin by increasing the mRNA levels of ANP, BNP, and *β*-MHC (Figure 7(a)). In addition, in PE induced cardiomyocyte hypertrophy, compared with treatment with diosmetin alone, the expression of HO-1 and SOD in ML385 and diosmetin cotreated cells was down-regulated, while the expression of p62 was also significantly downregulated (Figures 7(b) and 7(c)). These data directly suggest that ML385 eliminated the antioxidative stress effect of diosmetin. Taken together, the cardioprotective effect of diosmetin was associated with the activation of the Nrf2/HO-1 pathway (Figure 7(d)).

## 10. Discussion

In this study, we report that diosmetin is correlated with decreased autophagy and decreased ROS production, upon aortic constriction treatment in mouse. As diosmetin is known to alleviated stroke volume, ejection fraction, fractional shortening, LV hypertrophy and superoxide (O_2_·−) formation in high-fat diet-induced metabolic syndrome (MS) rats [24], our data indicate that diosmetin plays a protective role in myocardial remodeling by regulating of p62-Keap1-NRF2 signaling pathways and hence reducing ROS production.

In many experimental studies, diosmetin has been shown to regulate oxidative stress by modulating NRF2. For example, diosmetin inhibited Nrf2 and induced the production of reactive oxygen species (ROS) by inhibiting Nrf2 in ovarian cancer [19]. In non-small-cell lung cancer cells, diosmetin does not alter mRNA transcription of NRF2 but interferes with the stability of Nrf2 through Keap1-mediated proteasomal degradation [20]. This has not always been the case, in sepsis-induced acute kidney injury (AKI), the expression of the Nrf2/HO-1 signal axis was enhanced after diosmetin treatment, which were reversed by siNrf2 [26]. In addition, diosmetin also showed cardioprotective effects in MI model neonatal rats, accompanied by a decrease in p-Nrf2/Nrf2 [27]. Diosmetin protects against LPS-induced acute lung injury (ALI) via increasing the expression of Nrf2 along with its target gene HO-1 and blocked the activation of NLRP3 inflammasome both in vivo and in vitro [28]. However, the experimental results in this paper showed that during myocardial hypertrophy, no significant changes were found in the total Nrf2 expression levels in diosmetin-treated mice, but the nuclear Nrf2 levels increased, indicating that diosmetin can promote Nrf2 entry into the nucleus. This also explains the phenomenon that the expression of antioxidant genes increased in the diosmetin-treated group of mice after aortic constriction with unchanged total NRF2 expression.

As diosmetin has been confirmed to inhibit Akt activation in cancer cells including lung cancer cells and decrease p-AKT/AKT in MI model neonatal rats [29, 30], diosmetin dose-dependent reduced Akt phosphorylation and inhibited the PI3K/Akt pathway in non-small-cell lung cancer cells [20]. It was also found that treatment of hypoxia-injured cells with diosmetin alone promoted autophagy, whereas the results of WB analysis showed that AMPK signaling was activated by diosmetin. Diosmetin together with an autophagy inhibitor (3-methyladenine, 3-MA) or AMPK (compound C) was able to reduce diosmetin-induced autophagy as well as cytoprotective effects in hypoxia-injured cells [21]. In addition, autophagy-associated proteins mammalian target of rapamycin (mTOR), phosphatidylinositol 3-kinase, P70S6K, phosphoinositide-dependent kinase-1, extracellular signal-regulated kinase, 5′-AMP-activated protein kinase, and Akt were analyzed by western blotting, showing that diosmetin triggers autophagy by regulating the mTOR pathway in HepG2 cells [22]. As diosmetin was found to inhibit autophagy by activating upstream signaling pathway in our study (Figures 5(c) and 5(d)), the effect of diosmetin treatment on autophagy levels was assessed using mcherry-GFP-LC3 transfection and Western blotting analysis of autophagy-associated proteins. The autophagy inhibitor bafilomycin A1 was used to assess the relationship between diosmetin and cellular autophagy. This showed that autophagy inhibition is critical to diosmetin's protective effect in cardiac hypertrophy.

p62/SQSTM1 is an ubiquitin-binding autophagy receptor protein, linking the Nrf2 pathway to autophagy. p62 phosphorylation greatly enhances its affinity for Keap1, inducing the release of Nrf2 from Keap1 and the p62-Keap1 heterodimer recruits LC3 and mediates the permanent degradation of Keap1 in the selective autophagic pathway [31]. Ultimately, Nrf2 accumulates in the cytoplasm and then translocates to the nucleus, where it activates transcription of downstream genes encoding antioxidant enzymes, thereby protecting cells from oxidative damage. Since Nrf2 also upregulates the expression of the p62 gene, a p62-Keap1-Nrf2-positive feedback loop is formed, further enhancing the protective effect on cells [32, 33]. Accumulating evidence suggests that the p62-Keap1-Nrf2 pathway is critical in myocardial function and cardiac protection [34]. Thus, by maintaining the balance of the p62-Keap1-Nrf2-positive feedback loop as a bridge between the Nrf2 pathway and autophagy may be a potential target for the treatment of cardiac remodeling. Since autophagy inhibition by diosmetin led to the increase of p62 level, we hypothesized that noncanonical activation of Nrf2 by p62-Keap1 interaction may be the underlying mechanism behind the protective effect of diosmetin. In this study, we show that under oxidative stress conditions induced by aortic constriction, diosmetin enhances interaction between p62 and KEAP1 for sequestration and autophagic degradation of KEAP1. To further confirm that the protective effect of diosmetin on myocardial remodeling is dependent on the p62-Keap1-Nrf2 signaling pathway, the cardioprotective effect of diosmetin on PE-induced cardiac hypertrophy disappeared by knocking down the p62 gene in NRCMs cells. In contrast to previous studies of diosmetin in other diseases, the antioxidant effect of diosmetin during myocardial remodeling acts, at least in part, by modulating the cellular localization of Nrf2, rather than directly regulating Nrf2 expression. The reason for this discrepancy this may be due to different disease models. Taken together, our work highlights the importance of diosmetin in protein stabilization and suggests that therapeutic modulation of diosmetin may be a potential strategy for the treatment of protein disorders including cardiac remodeling.

## Data Availability

The research data used to support the findings of this study are included within the article.

## Supplementary Materials

Supplementary Materials Supplementary data Table 1: primer sequences for real-time PCR. Supplementary data Table 2: echocardiographic parameters of left ventricular function. (Supplementary Materials).
