# Supplementary material for: Diosmetin Protects against Cardiac Hypertrophy via p62/Keap1/Nrf2 Signaling Pathway
Source: Oxid Med Cell Longev. 2022 Feb 22;2022:8367997. doi: 10.1155/2022/8367997 (PMC8888112; doi:10.1155/2022/8367997)
Supplement: Supplementary Materials — Supplementary data Table 1: primer sequences for real-time PCR. Supplementary data Table 2: echocardiographic parameters of left ventricular function. (Supplementary Materials). [file 8367997.f1.docx]

Table 1 Primer sequences for Real-Time PCR


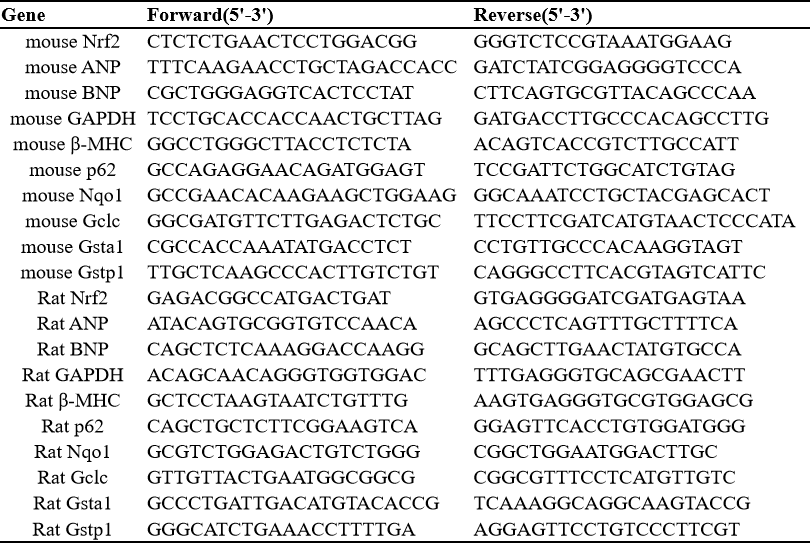


| **Table 2 Echocardiographic parameters of left ventricular function** | | | | |
| --- | --- | --- | --- | --- |
|  | **Sham** | | **AB** | |
| **Parameters** | **vehicle** | **Diosmetin** | **vehicle** | **Diosmetin** |
| **IVDd** | **0.632± 0.096** | **0.618±0.082** | **1.247±0.118***** | **0.923±0.092###** |
| **LVESd** | **2.33±0.333** | **2.45±0.251** | **4.283±0.371***** | **2.667±0.301###** |
| **edv** | **185.33±24.57** | **207.83±26.16** | **274.50±41.46***** | **205.50±25.46##** |
| **esv** | **52.83±13.56** | **57.17±10.26** | **144.67±24.29***** | **85.17±13.38###** |
| **sv** | **132.83±18.14** | **150.83±28.84** | **130.17±21.78** | **120.50±18.82** |

n = 6 in each group. Data are expressed as mean ± SD. ***p < 0.001 vs. vehicle group after sham operation. ##p < 0.01, ###p < 0.001 vs. vehicle group after AB operation. LVESd, Left ventricular end systolic diameter；IVDd，Left ventricular diastolic diameter；edv，End-diastolic volume；esv，end-systolic volume；sv，Stroke volume.
